# LC-MS/MS-based phytochemical profiling of *Erythrina caffra* leaves, isolation of a novel sterol glycoside, and evaluation of antioxidant, cytotoxic, and anti-sars-cov-2 activities

**DOI:** 10.1186/s13765-026-01126-w

**Published:** 2026-08-25

**Authors:** Bienvenu Tsakem, June Cheptoo Serem, Yvette Nkondo Hlophe, Nomusa Sikhakhane, Rémy Bertrand Teponno, Louis Pergaud Sandjo, Xavier Siwe Noundou

**Affiliations:** 1 Department of Pharmaceutical Sciences, School of Pharmacy, Sefako Makgatho Health Sciences University, PO Box 218 MEDUNSA, Pretoria, 0204 South Africa; 2 Department of Anatomy, School of Medicine, University of Pretoria, Pretoria, South Africa; 3 Department of Physiology, School of Medicine, University of Pretoria, Pretoria, South Africa; 4 Department of Chemistry, Faculty of Science, University of Dschang, Dschang, Cameroon; 5 Department of Chemistry, Federal University of Santa Catarina, Campus Universitário- Trindade, Florianópolis, 88040-900 SC Brazil

**Keywords:** *Erythrina caffra*, Cytotoxicity, Antioxidant activity, Anti-SARS-CoV-2 activity, Stigmasta-5,22-diene 3-*O*-(6'-tridecanoate)-β-*D*-glucopyranoside, LC-MS/MS analysis

## Abstract

From the crude ethanol extract of *Erythrina caffra* leaves, a new compound, stigmasta-5,22-diene 3-*O*-(6’-tridecanoate)-β-D-glucopyranoside **(1)**, along with stigmasta-5,22-diene (2), lutein **(3)**, and triacontanol **(4)**, was isolated. Their structures were elucidated using spectroscopic (1D and 2D NMR) and spectrometric [High-Resolution Electrospray Ionization Mass Spectrometry, (HRESIMS)] analyses. RP-LC-MS/MS profiling of the ethyl acetate and methanol fractions further identified 22 secondary metabolites, whose proposed structures were consistent with reported fragmentation patterns and literature data. The cytotoxic effects of the crude extract, fractions, and isolated compounds were evaluated on human keratinocytes (HaCaT), melanocytes (NHEM-Ad), and embryonic kidney cells (HEK293) via resazurin and crystal violet assays. Lutein **(3)** at 100 µg/mL significantly reduced cell viability to 39.19 ± 9.27%, 61.39 ± 3.26%, and 71.29 ± 7.82%, and cell density to 24.35 ± 4.29%, 35.96 ± 1.44%, and 39.16 ± 6.17%, respectively, for HaCaT, NHEM-Ad, and HEK293. In contrast, the crude extract and ethyl acetate fraction did not notably affect cell viability. Antioxidant evaluation revealed that the methanol fraction possessed the strongest activity (5.52 ± 0.14 µmol TE/mg) in the ORAC assay, while all isolated compounds and the crude extract exhibited significant peroxyl-radical scavenging activity. Plant-derived extracts and compounds showed weak to moderate inhibition of SARS-CoV-2 PLpro, with only the methanolic fraction exhibiting limited activity (IC₅₀<100 µg/mL), significantly lower than the reference inhibitor. Overall, these findings highlight the phytochemical diversity and biological potential of *E. caffra* leaves, emphasizing their antioxidant capacity and selective cytotoxicity. Future work should isolate and characterize the bioactive constituents responsible for the observed antioxidant effects.

**Graphical abstract:**

**Figure:**
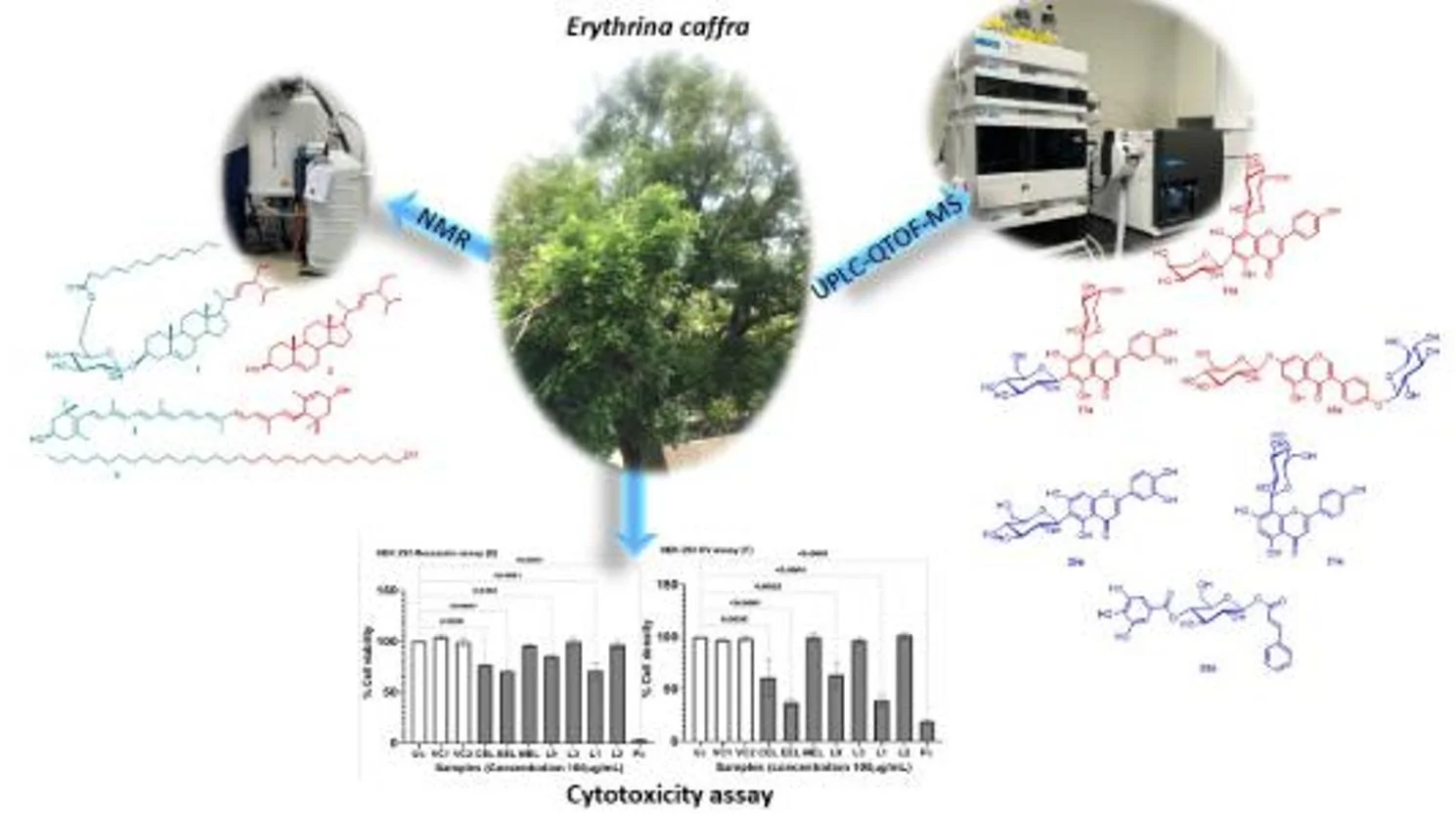

**Supplementary Information:**

The online version contains supplementary material available at 10.1186/s13765-026-01126-w.

## Introduction

The plant from the genus *Erythrina* (Fabaceae) are spread throughout the world, from Japan to America through Africa [1, 2]. Within this genus, *Erythrina caffra* is one of the well-known species growing in Africa, as the tree is widespread from South eastern Africa [3], to South Africa, where it is mainly located in the warm and frost-free to light frost regions [4]. The stem barks of the *E. caffra* are commonly used in traditional medicine to heal sores, wounds, arthritis, sprains and pains. Its leaves are ground into a paste and then used to relieve urinary infections and venereal diseases. The leaves are also infused and used as ear ache medicine [5]. Decoctions of the roots are used for sprains [6]. The diverse traditional uses of *E. caffra* for the treatment of wounds, infections, inflammatory conditions, and pain suggest the presence of bioactive secondary metabolites with potential pharmacological properties [5]. Previous phytochemical investigations of *Erythrina* species have revealed the occurrence of flavonoids, alkaloids, terpenoids, steroids, and other compounds exhibiting antioxidant, antimicrobial, cytotoxic, and antiviral activities [7–9]. Therefore, the evaluation of antioxidant, cytotoxic, and anti-SARS-CoV-2 activities is relevant for assessing the biological potential of *E. caffra* leaves and for identifying compounds that may contribute to its traditional medicinal applications. Some chemical studies already conducted on the stem bark of *E. caffra* led to the isolation of several prenylated flavonoids, one feruloyl acyl, steroids, one triterpenoid, and fatty alcohols [4, 7, 10]. As part of our continuous search for bioactive secondary metabolites from African medicinal plants [11, 12], the present study aimed to investigate the phytochemical composition and biological activities of the leaves of *E. caffra*. The ethanol extract was subjected to chromatographic separation to isolate and characterize its major constituents, while LC-MS/MS analysis was employed to profile the secondary metabolites present in the ethyl acetate and methanol fractions. The crude extract, fractions, and isolated compounds were subsequently evaluated for cytotoxic, antioxidant, and anti-SARS-CoV-2 PLpro activities. The findings contribute to the phytochemical knowledge of *E. caffra* and provide insight into the biological potential of its leaf constituents as sources of pharmacologically relevant natural products.

## Materials and methods

### General experimental procedures

¹H, ¹³C, and DEPT-135 NMR, as well as 2D NMR (¹H–¹H COSY, HSQC, HMBC) spectra, were recorded on a Bruker DRX-500 spectrometer operating at 400/500 MHz (¹H) and 100/125 MHz (¹³C) using deuterated solvents at 298 K; Chemical shifts (δ) are given in ppm relative to TMS as internal standard, and coupling constants (J) in Hz. Mass spectra were obtained by UPLC-QTOF-MS/MS in electrospray ionisation (ESI) mode. Column chromatography was performed using silica gel 60 (Merck, 70–230 and 230–400 mesh) and Sephadex LH-20 as stationary phases. TLC was carried out on silica gel 60 F₂₅₄ plates (Merck) and visualized under UV light (254/365 nm, Model UVGL-58, Upland, CA, USA) and/or after spraying with 10% H₂SO₄ followed by heating at 90 °C. Spectral data were processed using MestReNova, and LC–MS data were analyzed with MassLynx v4.2 software.

### Ultra-performance liquid chromatography QTOF-tandem mass spectrometry analysis of *E. caffra* extract

The ethyl acetate, and methanol fractions were subjected to qualitative analysis using an ultra-performance liquid chromatography quadrupole-time-of-flight mass spectrometer (UPLC-QTOF-MS) for identification of its chemical content. To carry out the analysis, the sample was prepared at a concentration of 1 mg/mL, for this purpose 1.0 mg of the samples were dissolved into 1 mL of MeOH. It was run on a Waters Acquity UPLC System equipped with a binary solvent delivery system and an autosampler. The column used was Waters BEH C18 (2.1 mm x 100 mm, 1.7 μm). As for the mobile phase, it consisted H_2_O + 0.1% formic acid (solvent A) and MeOH + 0.1% formic acid (solvent B), delivered in gradient mode; from 0 min to 0.10 min 3% of solvent B was applied, and gradually reached 100% at 14 min, and stayed constant up to 16 min, and finally this percentage was decreased to 3% from 16 to 16.5 min and stayed constant until 20 min. The flowrate was set at 0.3 mL/min. The injection volume was 5 µL.

The compounds resulting from the UPLC were analysed by a QTOF mass spectrometer, running in ESI (electrospray ionization) positive and negative modes. These compounds were tentatively identified by generating the respective molecular formula from MassLynx V 4.2; The lock mass compound was leucine enkephalin with *m/z* 554.2615 in negative mode and *m/z* 556.2771. The values of m/z were in accordance with the tolerance ranging from − 5.0 to 5 mDa; error ranging from − 15 to 5 ppm, and DBE: min = -1.5, max = 50.0. The corresponding structures were generated from the natural product database LOTUS and they were compare with published data first on the genus *Erythrina* and secondly in other plants; also, the fragmentation patterns was processed to support the structure of the identified compounds.

### Plant material

The leaves of *Erythrina caffra* were collected in May 2024 from the campus of Sefako Makgatho Health Sciences University, Ga-Rankuwa (Zone 1), Pretoria North, Gauteng Province, South Africa. A specimen of the plant was authenticated at SANBI by Mothogoane MS as *Erythrina caffra* Thunb., through comparison with a verified reference specimen bearing voucher number F. Olawale 1.

### Extraction and isolation

The plant material was dried and ground to yield 3.6 kg of powder, which was macerated three times (3 × 24 h) in 10 L of EtOH at room temperature. The filtrate obtained was then evaporated to dryness using rota vapour associating a vacuum pump to yield 202 g of crude extract. A mass of 200 g of the crude extract was suspended in distilled water (500 mL) and extracted with ethyl acetate (1000 mL) for liquid-liquid extraction. The ethyl acetate phase was collected 3 times and evaporated to dryness to yield ethyl acetate fraction (110 g). The aqueous residue was evaporated to dryness to yield the polar fraction and considered as MeOH fraction (86 g) (Figure S1).

A part of the EtOAc fraction (100 g) was adsorbed onto silica gel and then subjected to column chromatography using silica gel 60 (0.063–0.200 mm) eluted with *n*-hexane, and EtOAc 100% to afford seven sub-fractions LA (15.0 g), LB (18.0 g), LC (10 g), LD (25.0), LE (18.0 g) and LF (10.5 g). From LB compound **4** (2.0 g) precipitated as a white amorphous powder, and the filtrate was then evaporated and adsorbed onto silica gel and subjected to chromatography separation on silica gel, eluted with the mixture *n*-hexane: EtOAc (9:1) to lead to several sub-fractions (LB1-LB5). From the sub-fraction LB3, compound **2** (230.0 mg) precipitated as white amorphous powder. The sub-fraction LE equally undergone a separation on silica gel column chromatography, with the mixture of *n*-hexane: EtOAc (7:3) as eluent to afford the sub-fractions LE1-LE7; Compound **3** (326.1 mg) precipitated as red powder. As for the sub-fraction LF, it was subjected to separation on column chromatography using silica gel as the stationary phase and eluted with *n*-hexane: EtOAc (3:2), four sub-fraction resulted from the fractionation; and compound **1** (32.0 mg) precipitated as white gum (Figure S1).

### Total phenolic content of the crude extract and fractions

The principle of total phenolic content (TPC) is based on the reduction of the F–C reagent in an alkaline medium by phenolic compounds. This reduction produces a blue-coloured complex with maximum absorbance around 630. The absorbance is directly proportional to the phenolic content.

The TPC of the *E. caffra* crude extract and fractions was determined using the Folin–Ciocalteu (F-C) colorimetric method. The study followed the method reported by Molole and coworkers [13] with modifications. Briefly, appropriate concentrations of the samples were prepared in phosphate-buffered saline (PBS) PBS: Dimethyl sulfoxide (DMSO) (9:1), and MeOH: HCl (99:1) (same solvent system for dissolution of sample in the biological assays). Then they were diluted with pure PBS to 1% and 0.1% of DMSO and HCl. This was to maintain consistency and enable a direct comparison between phenolic content and biological effects. This approach was adopted to mimic physiologically relevant conditions. Thereby ensuring that the measured phenolic content could be meaningfully linked to the antioxidant and cytoprotective effects observed in vitro, thus reflecting the effective concentrations likely to be encountered in biological systems. The stock solutions were then prepared at 1 mg/mL, and gallic acid standards (0, 0.04, 0.08, 0.16, 0.2 mg/mL) were prepared in distilled water. For the assay, 10 µL of each extract or standard was mixed with 50 µL of diluted F–C reagent (1:10 with water), followed by the addition of 50 µL of 7.5% w/v Na₂CO₃ solution. The blank wells contained 10 µL of distilled water and 50 µL of each of the aforementioned solutions. The mixtures were incubated at room temperature for 30 min. And the absorbance was measured at the optimized wavelength of 630 nm. The calibration curve was constructed using gallic acid standards (0–150 µg/mL), which showed excellent linearity (R² = 0.9981) (Figure S2) with a y-intercept set at zero. The assay was performed in three different repeats. For each replicate, the active concentrations of the samples were calculated from the calibration curve using equation (Eq. 1). The corresponding effective masses were then obtained by applying equation (Eq. 2**)**, where *V* represented the total reaction volume in each well (110 µL). Finally, the TPC values were expressed as milligrams of gallic acid equivalents (GAE) per gram of dry sample by dividing the calculated phenolic mass by the actual mass of the sample present in the 10 µL aliquot introduced into each well.

1 *y=mx*

With y representing the absorbance of each sample, x represents the equivalent concentration in mg/mL, and m represents the gradient or slope

2 $$GAE=x \times \frac{v}{m}$$

With m = the mass of the sample in g (m = 0.00001 g), V = the total volume of the well in mL.

### Antioxidant capacity

The antioxidant capacity of the extracts, fractions, and isolated compounds was evaluated using the Oxygen Radical Absorbance Capacity (ORAC) assay. The ORAC assay relies on a hydrogen atom transfer (HAT) mechanism, in which peroxyl radicals oxidize fluorescein, resulting in fluorescence decay that reflects antioxidant activity over time. By contrast, the ABTS assay follows a single electron transfer (SET) mechanism, where antioxidants donate electrons to reduce the ABTS•⁺ radical cation, leading to loss of its blue-green coloration [14].

### Oxygen radical absorbance capacity assay

In this method, 2,2′-azobis(2-amidinopropane) dihydrochloride (AAPH, 8 mg/mL) served as a free radical initiator to generate peroxyl radicals, while fluorescein (final concentration: 1.39 × 10⁻⁶ M) was used as the fluorescent probe. A Trolox standard curve was prepared over a concentration range of 0–1 mM for reference.

For the assay, 10 µL of each sample (1 mg/mL) was added to 165 µL of fluorescein solution in a 96-well black plate, followed by 25 µL of AAPH solution, resulting in a final sample concentration of 50 µg/mL per well. Fluorescence was monitored at 485 nm excitation and 520 nm emission using a FLUORstar OPTIMA plate reader (BMG Labtech, Offenburg, Germany) maintained at 37 °C for 2 h. The fluorescence decay data were integrated to calculate the area under the curve (AUC) using Microsoft Excel. Antioxidant activity was determined relative to the Trolox calibration curve and expressed as micromoles of Trolox equivalents per milligram of sample (µmol TE/mg).

### 2,2′-Azino-bis(3-ethylbenzothiazoline-6-sulfonic acid)/ABTS assay

For this second mechanism of assessing the antioxidant capacity, Trolox was once again used as the standard antioxidant. It was prepared in the same concentration range as in the ORAC assay (0–1 mM). The ABTS·⁺ radical solution was freshly prepared 12 h before analysis by dissolving 0.219 g of ABTS in 5 mL of 0.1 M PBS and mixing it with 5 mL of 0.1 M PBS containing 0.0041 g of potassium persulfate. The resulting mixture was kept in the dark at room temperature for 12 h to ensure complete generation of the ABTS·⁺ radical. For the assay, 10 µL of each test sample (1 mg/mL) was pipetted into individual wells of a 96-well plate, followed by 290 µL of the ABTS·⁺ solution. Control wells contained only the ABTS·⁺ solution and distilled water. The plate was incubated in the dark at room temperature for 15 min to allow the reaction to occur, after which the absorbance was recorded at 734 nm using a UV-Vis microplate reader. Antioxidant activity was calculated from the Trolox calibration curve and expressed as micromoles of Trolox equivalents per milligram of sample (µmol TE/mg).

### Absorption, distribution, metabolism, excretion and toxicity properties

Pharmacokinetics refers to the study of the potential fate of a therapeutic compound within the body, and it plays a crucial role in drug discovery and development. The individual indices implicated in this pharmacokinetic include ADMET. For this purpose, the chemical structures were drawn from Chemdraw, then the smiles were generated and loaded online in the ADMETlab 3.0 software (https://admetlab3.scbdd.com).

### In vitro cytotoxicity assessment

HaCaT, NHEM-Ad, and HEK 293 cells were maintained in DMEM supplemented with 10% fetal calf serum and 1% antibiotic/antimycotic mixture (10,000 U penicillin, 10 mg streptomycin, 25 µg amphotericin B per mL), at 37 °C in a humidified incubator with 5% CO₂ until reaching 70–80% confluence. Cells were detached using 0.05% trypsin (for HaCaT and NHEM-Ad) or TrypLE™ Express (for HEK 293), counted, and seeded into 96-well plates at a density of 10 × 10⁴ cells per well in 90 µL medium, then allowed to adhere overnight. The following day, cells were treated with 10 µL of test samples to reach a final concentration of 0.1 mg/mL for 24 h. Controls included PBS (negative), 1% Triton X-100 (positive), and vehicles (0.1% DMSO or 0.01% HCl in methanol). Cytotoxicity was assessed using resazurin and crystal violet assays.

### Resazurin assay

After the exposure period, 11 µL of resazurin solution (prepared in 0.1 M PBS) was added to each well for the assay. The plates were then incubated for an additional 3 h for HaCaT and NHEM-Ad cells, and 6 h for HEK 293 cells. Following incubation, fluorescence was measured using a FLUOR microplate reader (Germany) equipped with Omega software, at an excitation wavelength of 544 nm and an emission wavelength of 590 nm [15]. The obtained fluorescence values were used to calculate the percentage of cell viability, with results expressed relative to the negative control, representing 100% cell viability.

### Crystal violet assay

Following fluorescence measurement in the resazurin assay, cells were fixed by adding 11 µL of 20% (v/v) formaldehyde to reach a final concentration of 2%, followed by a 30-minute incubation at room temperature. The wells were rinsed with tap water and air-dried. Subsequently, 100 µL of 0.1% (w/v) crystal violet solution was added to each well and incubated for 1 h to allow staining. Excess dye was washed off with tap water, and the plates were left to dry completely. Cell morphology was then examined and photographed under a Zeiss microscope [15]. To quantify cell density, the bound dye was solubilized with 100 µL of 10% (v/v) acetic acid, and absorbance was measured at 630 nm using a FLUOR microplate reader (Germany). Data were expressed as percentage cell density relative to the negative control.

### *Anti-SARS-CoV-2 activity*: In vitro PLpro protease inhibition assay

The inhibitory activity of crude extracts, fractions, and isolated compounds against SARS-CoV-2 was evaluated using a fluorescence-based assay on a GloMax^®^ Explorer microplate reader. It was evaluated using the Papain-like Protease (SARS-CoV-2) Assay Kit (BPS Bioscience, USA; Cat. No. 79995-1). The assay employed a recombinant SARS-CoV-2 PLpro enzyme and the fluorogenic substrate Z-RLRGG-AMC (Z-Arg-Leu-Arg-Gly-Gly-7-amido-4-methylcoumarin). The enzyme was used at a concentration of 0.3–0.5 ng/µL (9–15 ng per reaction), while the substrate was used at a final concentration of 25 µM. Enzymatic activity was monitored through the release of AMC fluorescence following substrate cleavage. The enzyme was pre-incubated with test samples, reference inhibitor (GRL0617), or buffer at 37 °C for 30 min, followed by addition of a fluorogenic substrate. Fluorescence was monitored at excitation/emission wavelengths of 495–505/365 nm after 12 h incubation.

Test samples were prepared in PBS: DMSO (9:1) or PBS: MeOH (90:9.9) with 0.01% HCl and assayed at final concentrations ranging from 0.1 to 0.8 mg/mL. Experimental conditions included positive control, inhibitor control, blank, and test samples. Enzyme inhibition was calculated relative to the inhibitor control, and IC₅₀ values were determined to assess antiviral activity (Eq. 3).

3 $$\% activity= \frac{Fluorescence_{sample} - Fluorescence_{blank} }{Fluorescence_{inhabitor\,control- Fluorescence_{blank}}} \times 100$$

### Statistics

All experiments were conducted with a minimum of two technical replicates and three independent biological repeats to guarantee the reproducibility of the tests. Quantitative results are presented as mean ± standard error of the mean (SEM). Data normality was assessed using the Shapiro–Wilk test. For statistical analysis, one-way ANOVA followed by Tukey’s post hoc test was applied to datasets with a normal distribution (parametric). In cases where the data were nonparametric, the Kruskal–Wallis test was followed by Dunn’s multiple comparisons test. A p-value ≤ 0.05 was considered statistically significant.

## Results and discussion

### Structure elucidation of isolated compounds

The leaves of *Erythrina caffra* were extracted with EtOH and the purification of the crude extract afforded the unreported stigmasta-5,22-diene 3-*O*-(6’-tridecanoate)-β-*D*-glucopyranoside (**1**), together with stigmasterol (**2**) [3], lutein (**3**) [16], and triacontanol (**4**) [17].

Compound **1** was obtained as a brown gum, and its FTIR spectrum (Figure S3) exhibited bands at 1740, 2929, 3100–3600 cm^− 1^, corresponding to C = O, Csp^2^-H and OH-groups, respectively. Its HRESIMS(+) spectrum (Figure S4 and S5) showed the sodium adduct [M + Na]^+^ at *m*/*z* 793.5988 (calcd for C_48_H_82_O_7_Na^+^: 793.5953) indicative of the molecular formula C_48_H_82_O_7_, with 8 unsaturations.

The ^1^H NMR spectrum (Figure S6) exhibited some resonances at *δ*_H_ 0.72 (s, H-18), 1.03 (o, H-19), 1.04 (o, H-21), 0.79 (d, *J* = 6.1 Hz, H-26), 0.82 (o, H-27) and 0.81 (o, H-29) attributable to methyl groups characterising stigmasterol-like structure; this was supported by the olefinic protons displayed at *δ*_H_ 5.38 (d, *J* = 5.1 Hz, H-6), 5.18 (dd, *J* = 15.1, 8.6 Hz, 1H), and 5.05 (dd, *J* = 15.2, 8.5 Hz, 1H) (Table 1) characterising the Δ^5(6)^ and Δ^22(23)^ double bonds of stigmasterol [18]. Moreover, the signals observed at *δ*_H_ 0.88 (d, *J* = 6.2 Hz, H-13’’) together with the intense signal in the range 1.25–1.31 (m, H-4’’ to H-9’’) suggested a fatty acid moiety. Further examinations of the spectrum revealed certain signals at *δ*_H_ 4.40 (o, H-1’), 3.39 (o, H-2’), 3.57 (t, *J* = 7.0 Hz, H-3’), 3.48 (br.d, H-4’), 3.40 (o, H-5’), 4.42 (o, H-6’a), and 4.33 (dd, *J* = 12.2, 2.3; H-6’b), attributed to one β-glucopyranoside unit.

From the interpretation of ^13^C NMR and DEPT 135 spectra (Figure S7 and S8) of compound 1, the signals at *δ*_C_ 101.2 (C-1’), 73.6 (C-2’), 76.1 (C-3’), 70.3 (C-4’), 74.0 (C-5’) and 63.3 (C-6’) supported the presence of β-glucopyranoside moiety [19]. However, the other signals exhibited at *δ*_C_ 174.4 (C-1’’), 34.3 (C-2’’), 25.0 (C-3’’), 29.5–29.8 (C-4’’ to C-9’’), and 13.9 (C-13’’) supported the presence of a fatty acid fragment. The Heteronuclear Single Quantum Correlation (HSQC) spectrum (Figure S9) of compound **1** enabled appropriate attribution of proton signals to their respective carbons [20] such as the correlation from the proton at *δ*_H_ 3.55 (H-3) to the carbon at *δ*_C_ 79.6 (C-3), as well as the correlation from the two diastereostopic protons at *δ*_H_ 4.42 (H-6’a) and 4.33 (H-6’b) to the carbon at 63.3 (C-6’); however, these data suggested an etherification of C-3 of stigmasterol and the esterification of C-6’. These assertions were evidenced from the Heteronuclear Multiple Bond Correlation (HMBC) experiment (Figure S10), which assisted in establishing the correlations from protons to carbons indirectly bonded. The correlation from the proton at *δ*_H_ 3.55 (H-3) to the carbon at *δ*_C_101.2 (C-1’), then from *δ*_H_ 4.40 (H-1’) to *δ*_C_79.6 (C-3), supported the fixation of β-*D*-glucopyranosyl on the stigmasteryl structure. Furthermore, the correlation from the protons at *δ*_H_ 4.42 (H-6’a) and 4.33 (H-6’b) to the carbon at *δ*_C_174.4 (C-1’’) justified the linkage of the fatty acid chain on the C-6’’ of β-*D*-glucopyranosyl. However, the number of carbon atoms of the fatty acid moiety was confirmed via the fragment ion exhibited at *m/z* 169.2005 corresponding to C_12_H_25_^+^ (Figure S11).

**Fig. 1:**
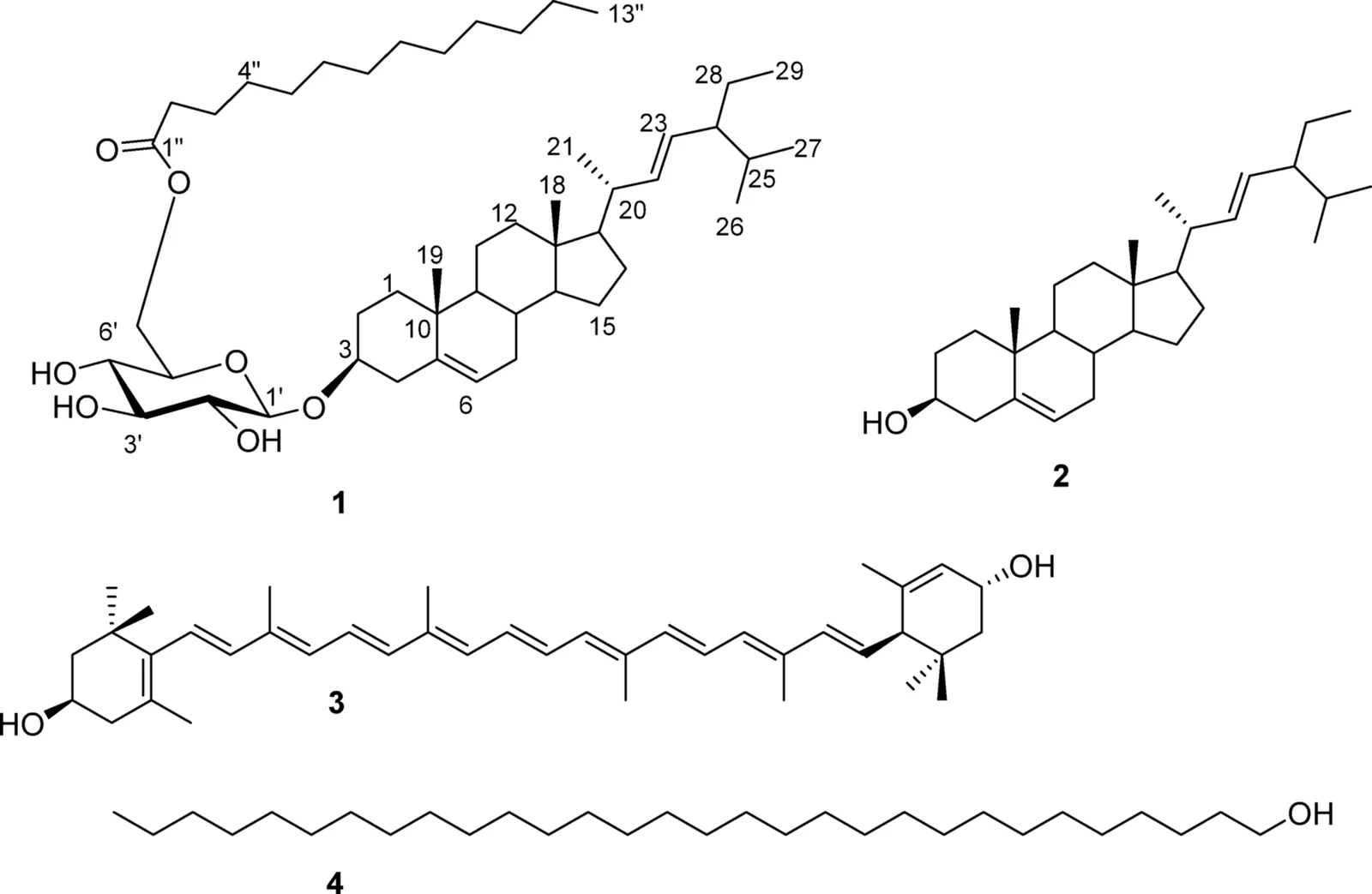
Chemical structures of compounds **1–4**

Therefore, the combination of ^1^H and ^13^C NMR, HSQC, HMBC, and ^1^H-^1^H COSY (Figure S12) and MS (Figures S13-S15) data in comparison to those reported in the literature led to elucidation of compound **1** as the novel stigmasta-5,22-diene 3-*O*-(6’-tridecanoate)-β-*D*-glucopyranoside, trivially named erythrinoside (Fig .1)

**Table 1:** ^1^H NMR (CDCl_3_, 400 MHz) and ^13^C NMR (CDCl_3_, 100 MHz) data of **1**

Position	δ_C_	δ_H_ (multiplicity, J in Hz)
1	37.2	1.86 (m, H-1a); 1.03 (m, H-1b)
2	39.7	2.00 (m, H-2a); 1.17 (m, H-2b)
3	79.6	3.55 (o, H-3)
4	38.9	2.38 (o, H-4a); 2.26 (o, H-4b)
5	140.5	/
6	122.2	5.38 (d, *J* = 5.1 Hz, H-6)
7	29.3	1.94 (m, H-7a); 1.63 (o, H-7b)
8	31.9	1.48 (m, H-8)
9	50.2	0.94 (o, H-9)
10	36.8	/
11	21.3	1.24 (m, H-11a); 1.15 (m, H-11b)
12	39.7	2.02 (m, H-12a); 1.20 (m, H-12b)
13	42.2	/
14	56.0	1.08 (m, H-14)
15	24.3	1.51 (m, H-15a); 0.98 (m, H-15b)
16	29.2	1.64 (m, H-16a); 1.13 (m, H-16b)
17	56.9	0.95 (o, H-17)
18	12.0	0.72 (s, H-18)
19	20.9	1.03 (o, H-19)
20	40.4	2.07 (m, H-20)
21	21.2	1.04 (o, H-21)
22	138.2	5.18 (dd, *J* = 15.1, 8.6 Hz, 1 H)
23	129.3	5.05 (dd, *J* = 15.2, 8.5 Hz, 1 H)
24	51.1	1.54 (m, H-1a)
25	31.8	1.63 (o, H-25)
26	21.1	0.79 (d, *J* = 6.1 Hz, H-26)
27	19.7	0.82 (o, H-27)
28	24.8	1.02 (o, H-28a); 1.01 (o, H-28b)
29	12.2	0.81 (o, H-29)
1’	101.2	4.40 (o, H-1’)
2’	73.6	3.39 (o, H-2’)
3’	76.1	3.57 (t, *J* = 7.0 Hz, H-3’)
4’	70.3	3.48 (br.d, H-4’)
5’	74.0	3.40 (o, H-5’)
6’	63.3	4.42 (o, H-6’a); 4.33 (dd, *J* = 12.2, 2.3; H-6’b)
1’’	174.4	/
2’’	34.3	2.36 (o, H-2’’)
3’’	25.0	1.63 (o, H-3’’)
4’’-9’’	29.5–29.8	1.25–1.31 (m, H-4’’ to H-21’’)
11’’	31.3	1.31 (o, H-22’’)
12’’	22.4	1.31 (o, H-23’’)
13’’	13.9	(d, *J* = 6.2, H-24’’)

d = doublet, o = overlapped, m = multiplet, br.d = broad doublet

### UPLC-MS/MS analysis of *Erythrina caffra* leaves extract

**Fig. 2:**
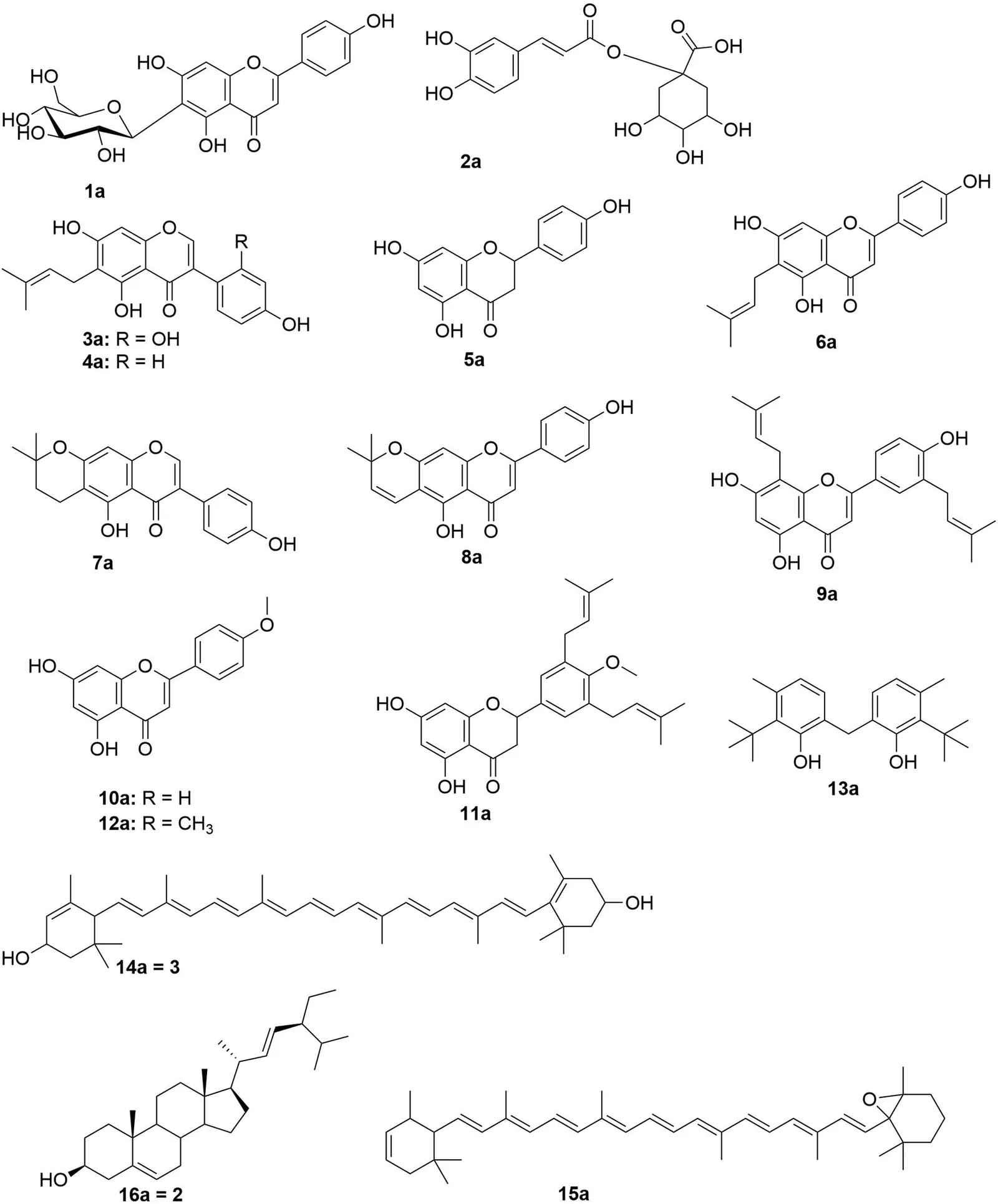
Chemical structures of some identified compounds by LC-MS/MS from ethyl acetate fraction of *E. caffra* leaves extract

**Fig. 3:**
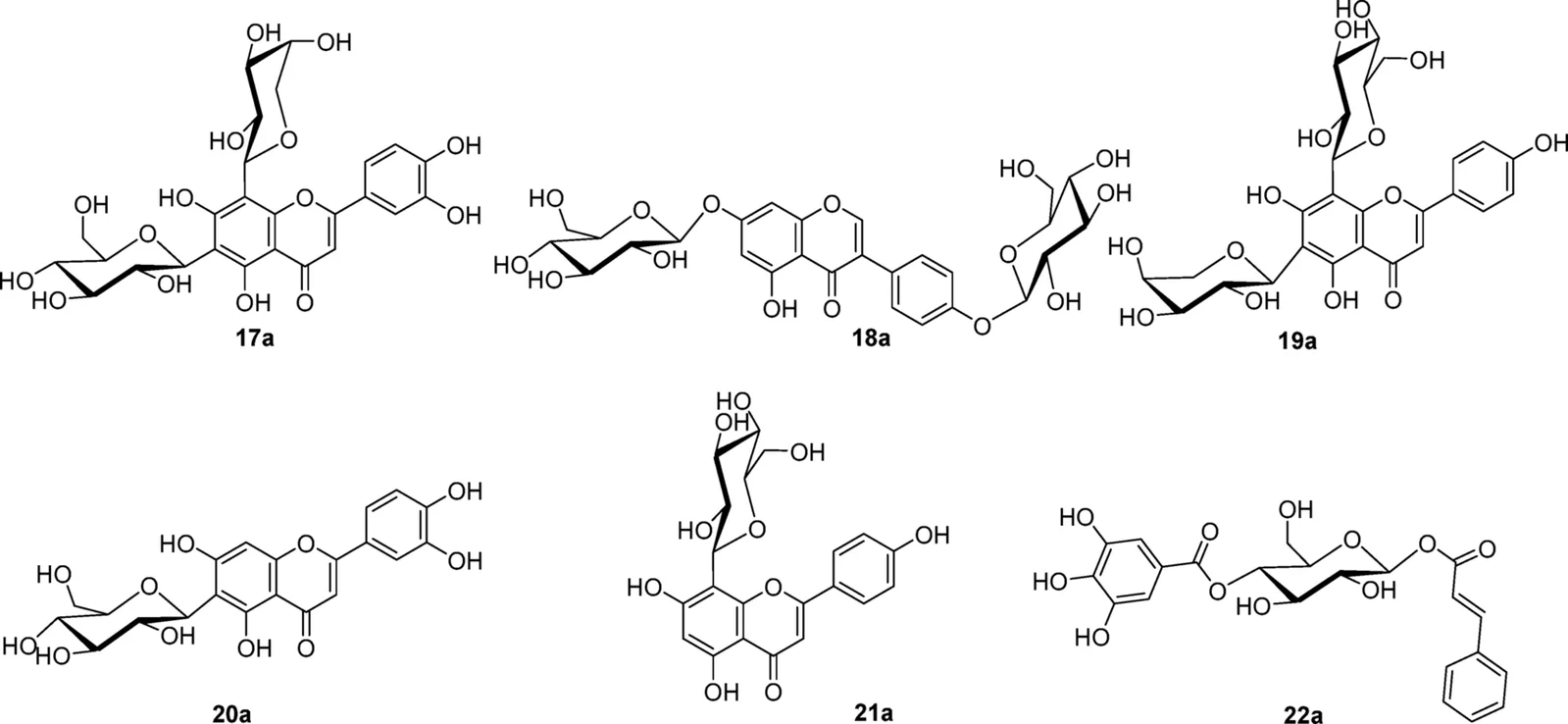
Chemical structure of some identified compounds by LC-MS/MS from MeOH fraction of *E. caffra* leaves

The metabolite profile of the EtOAc and MeOH fractions of *E. caffra* leaves was performed through RP-LC-MS/MS in positive and negative ESI ionization modes (Figure S19-S21). Based on molecular formulas predicted by MassLynx, the structures were matched to entries in the LOTUS [21], ChemSpider, and PubChem databases. The ethyl acetate fraction yielded sixteen putative metabolites (Fig. 2), while six were identified in the methanol fraction (Fig. 3) according to their retention profiles. For these compounds, tentative names were proposed (Table 2). A fragmentation pattern [22] was proposed for each compound (Figures S22 and S23) to support the proposed structures, and according to the molecular formula generated through MS-MS in MassLynx.

**Table 2:** LC-MS/MS metabolite profiling of the EtOAc and methanol fractions of E. caffra leaves

N°	RT	Adducts and m/z ratio	Molecular formula and RMM	Fragment ions in ESI(+) and ESI (-)	Tentative names, plant sources and references
1a	7.94	[M-H+HCOOH]^-^m/z477.1073	C_22_H_21_O_12_^-^477.1038	317.0844 (+), 121.0268 (+), 269.0459 (-)	Isovitexin ( Gentiana arisanensis) [23]
2a	8.34	[M-H+HCOOH]^-^m/z399.0944	C_17_H_19_O_11_^-^399.0933	165.0377 (+), 117.0327 (+)135.0414 (-), 129.0542 (-), 109.0276 (-)	Chlorogenic acid ( Erythrina variegata) [24]
3a	10.09	[M+H]^+^m/z355.1165[M-H]^-^353.1013	C_20_H_19_O_6_^+^355.1176C_20_H_17_O_6_^-^353.1031	299.0515 (+), 137.0223 (+)137.0582 (+),165.0170 (+)	2,3-Dehydrokievitone ( Erythrina sacleuxii) [25]
4a	11.49	[M+H]^+^m/z339.1230	C_20_H_19_O_5_^+^339.1227	283.0577 (+), 165.0170 (+), 221.0853 (+), 139.0408 (+), 195.0984 (+), 119.0483 (+), 137.0233 (+),161.0218 (-), 117.0326 (-), 133.0271 (-), 193.0832 (-), 147.0424 (-)	6-prenylgenistein/wighteone ( E. indica) [26]
5a	11.50	[M+Na]^+^m/z295.0568	C_15_H_12_O_5_Na^+^295.0577	121.0273, 281.0420	Naringenin ( Dalbergia parviflora) [27]
6a	11.52	[M+H]^+^m/z339.1230	C_20_H_19_O_5_^+^339.1232	165.0170, 283.0577, 147.0425 (-), 117.0326 (-),161.0218 (-), 193.0831 (-)	6-prenylapigenin ( Erythrina vogelii) [28]
7a	11.69	[M+H]^+^m/z339.1217	C_20_H_19_O_5_^+^339,1227	283.0578, 221.0787, 165.0182, 137.0215	Erythrivarone A ( Erythrina variegate) [29]
8a	12.55	[M+Na]^+^m/z359.0864[M-H]^-^337.1076	C_20_H_16_O_5_Na^+^359.0890	191.0675 (-), 161.0229 (-)147.0426 (-)	Carpachromene ( Erythrina vogelii) [2]
9a	13.04	[M+Na]^+^m/z429.1660[M-H]^-^m/z405.1690	C_25_H_26_O_5_Na^+^429.1672C_25_H_25_O­_5_^-^405.1707	351.1205, 295.0579, 177.0166, 165.0178	Gancaonins Q ( Gycyrrhiza uralensi) [30]
10a	13.48	[M+H]^+^m/z285.0735	C_16_H_13_O_5_^+^285.0757	177.0525 (+), 153.0179 (+)155.0304 (+)	Acacetin ( Calea urticifolia) [31]
11a	13.69	[M-H]^-^m/z421.2000	C_26_H_29_O_5_^-^421.2020	125.0199 (-), 299.1989 (-)	Abyssinone V 4′-methyl ether (Erythrina caffra) [4]
12a	13.72	[M+H]^+^m/z299.0936	C_17_H_15_O_5_^+^299.0914	167.0345 (+), 161.0562 (+)	Apigenin-7,4’-dimethyl ether (Plectranthus ruticosus) [32]
13a	14.04	[M-H]^-^m/z339.2310	C_23_H_31_O_2_^-^339.2310	163.1106 (-)	2,2'-methylenebis[6-ter-butyl-4-methylphenol] (Senna italica) [33]
14a	14.32	[M+H]^+^m/z569.4314	C_40_H_57_O_2_^+^569.4353	139.1107 (+), 153.1261 (+),549.4079 (+), 429.3170 (+)	Lutein (Chlorella sorokiniana) [34]
15a	15.23	[M+Na]^+^m/z575.4231	C_40_H_56_ONa^+^575.4223	139.1094 (+), 137.1308 (+)153.1256 (+), 429.3163 (+)	β-Carotene 5,6-epoxide (Telekia speciosa) [35]
16a	16.3	[M+H]^+^m/z413.3781	C_29_H_49_O^+^413.3778	213.1621 (+), 255.2083 (+), 395.3646 (+), 297.2606 (+)	Stigmasterol (Erythrina caffra) [3]
17a	5.36	[M-H]^-^,m/z579.1353	C_26_H_27_O_15_^-^579,1355	489.1035(-), 459.0923(-), 235.0228(-), 133.0309(-)	Lucenin III (Ephedra alata), [36]
18a	5.63	[M-H]^-^,m/z593.1512	C_27_H_29_O_15_^-^593.1512	431.0984(-)	Genistein 4,7’-diglucopyranoside (Thermopsismontana)[37]
19a	6.44	[M-H]^-^,m/z563.1399	C_26_H_27_O_14_^-^563.1406	473.1071(-), 443.0966 (-)	Isoschaftoside (Erythrina abyssinica), [38]
20a	6.61	[M-H]^-^,m/z447.0937	C_21_H_19_O_11_^-^447.0933	357.0592 (-), 299.0567(-), 285.0408 (-)	Luteolin-6-C-β-D-glucopyranoside (Erythrina crista galli) [39]
21a	6.72	[M-H]^-^,m/z431.0967	C_21_H_19_O_10_^-^ 431.0984	313.0597(-), 311.0540(-), 117.0322 (-)	Vitexin (Erythrina speciosa) [40]
22a	6.89	[M-H]^-^,m/z461.1074	C_22_H_21_O_11_^-^461.1089	293.1033(-), 161.0127(-), 125.0227(-)	1-O-(E)-cinnamoyl-4-galloyl-β-D-glucopyranose (Balanophora fungosa) [41]

**Fig. 4:**
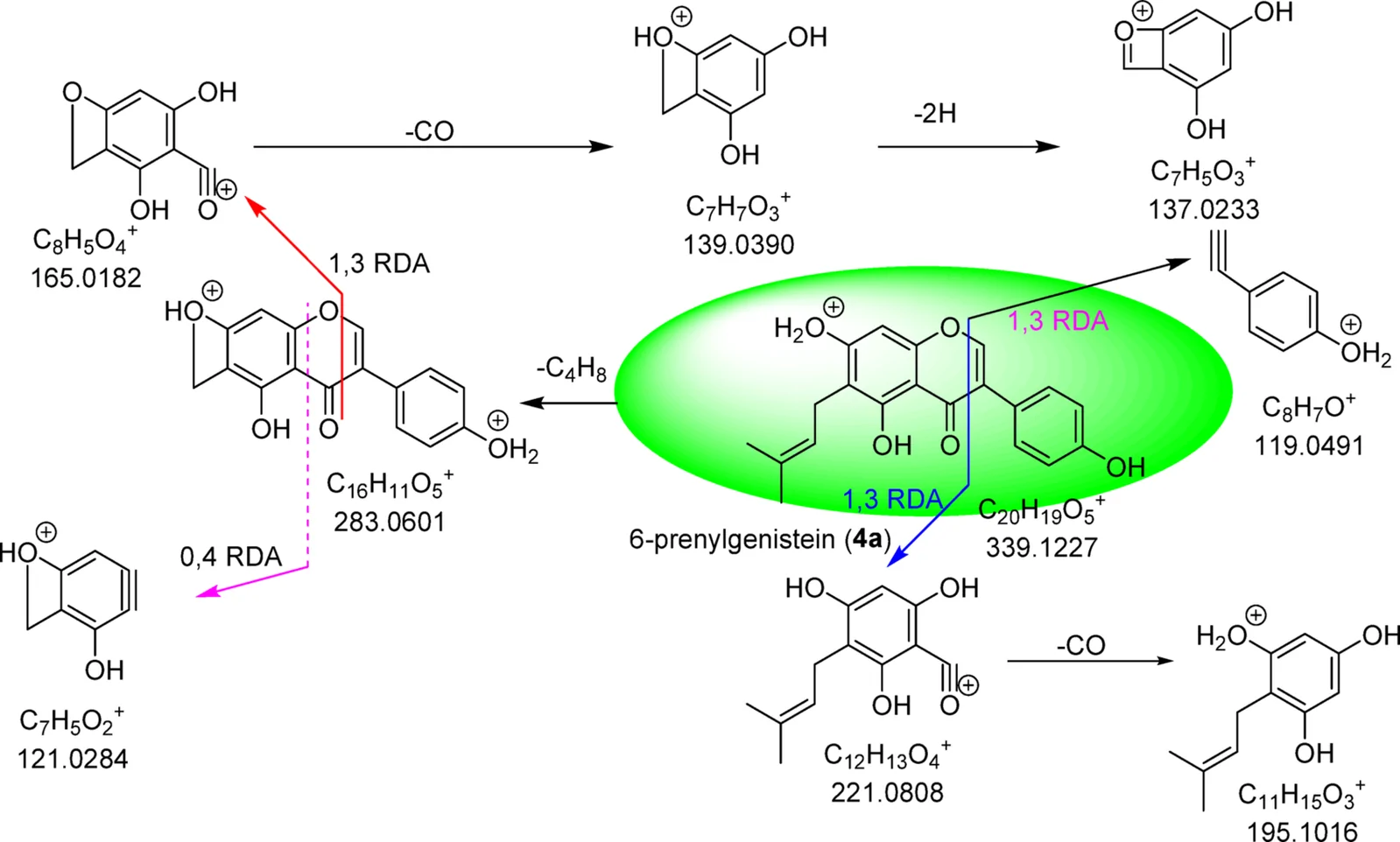
Fragmentation mechanism of 6-prenylgenistein (**4a**) in HRESIMS/MS(+), HRESIMS/MS: High Resolution Electrospray ionisation Mass Spectrometry/Mass Spectrometry, RDA: Retro Diels-Alder

**Fig. 5:**
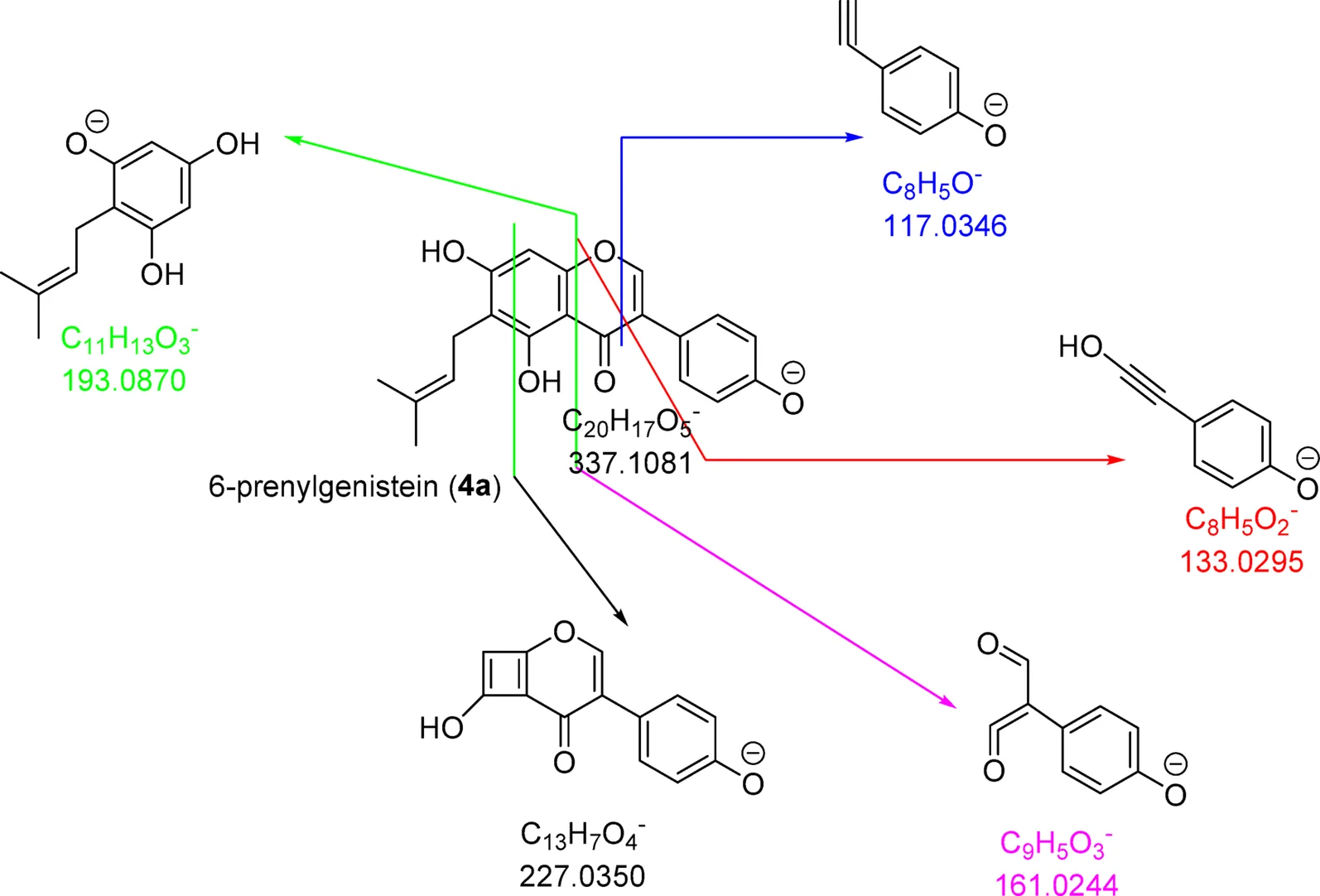
Fragmentation mechanism of 6-prenylgenistein (**4a**) in HRESIMS/MS(-) HRESIMS/MS: High Resolution Electrospray ionisation Mass Spectrometry/Mass Spectrometry, RDA: Retro Diels-Alder

Figures 4 and 5 show a fragmentation pattern of 6-prenylgenistein (4a) in both positive and negative modes [22]. In MS analysis, compound 4a fragmented mainly via retro-diels alder (RDA) which easily affect the prenyl group attached on the ring A to lead to the fragment at m/z 283 appearing as the most intense fragment ion, several other RDA mechanisms occur in the C-ring and lead to 1,3; 1,4 fragments such as the fragment ions at *m/z* 165 and 119 resulting from 1,3 RDA cleavage of 4a. According to the report of Lewars and co-workers, the fragment at *m/z* 121 derives from the 0,4 RDA [42]. Further rearrangements of RDA resulting fragment ions lead to elimination of CO and H_2_. Figure 4 shows the LC-MS/MS of 4a in ESI positive mode.

In ESI negative mode, the fragmentation equally follow the RDA, the common fragment ions include the two fragments resulting from 1,3 cleavage [43, 44]. Other cleavages include 1,3 and 0,4 RDA cleavages [44] (Fig. 5). *C*-glycosyl flavones fragment mainly through cross-ring cleavages of the sugar moiety (rather than simple sugar loss as in *O*-glycosides). Diagnostic ions are produced at m/z losses of 90, 120, and 150 Da, corresponding to C_3_H_6_O_3_, C_4_H_8_O_4_, and C_5_H_10_O_5_ eliminations from the hexose (Figure S23) [45]. *C*-diglycosides Show additional fragmentation with sequential sugar losses (162, 132, 146 Da depending on sugar type) (Figure S23), still dominated by cross-ring cleavages [46].

Interestingly, 11 compounds out of 22 identified were isolated previously from the genus *Erythrina*. Chlorogenic acid (**2a**) was obtained from the stem bark of *Erythrina variegata* [24], the chemical studies of the stem bark extract of *Erythrina sacleuxii* previously led to 2,3-dehydrokievitone (**3a**) [25], 6-prenylgenistein or commonly known as wighteone (**4a**) was isolated from the wood of *Erythrina varieta* [1] and from the stem bark of *E. indica* [26]. From the stem bark of the west African plant *Erythrina vogelii*, 6-prenylapigenin (**6a**) was isolated [28], Erythrivarone A (**7a**) was characterized from the stem bark of *Erythrina variegata* [29]. Ali and co-workers investigated the leaves of the Cameroonian plant *Erythrina vogelii* and obtained Carpachromene (**8a**) amongst other compounds [2], whereas abyssinone V 4′-methyl ether (**11a**) is commonly isolated from several *Erythrina species*, including *Erythrina caffra* [4]. Stigmasterol (**16a**) was equally isolated from *Erythrina caffra* [3]. Other compounds including Naringenin (**5a**), gancaonin Q (**9a**) [30], acacetin (**10a**), and 2,2’-methylenebis[6-ter-butyl-4-methylphenol] (**13a**) were all previously isolated from the Fabaceae family. From the reported data, isoschaftoside (**19a**) has been isolated from the stem barks of *Erythrina abyssinica* [38], luteolin-6-*C-*β*-*D-glucopyranoside (**20a**) was reported from the leaves of *Erythrina crista galli* [39], and vitexin (**21a**) was isolated from the leaves of *Erythrina speciosa* [40].

### Total phenolic content

**Fig. 6:**
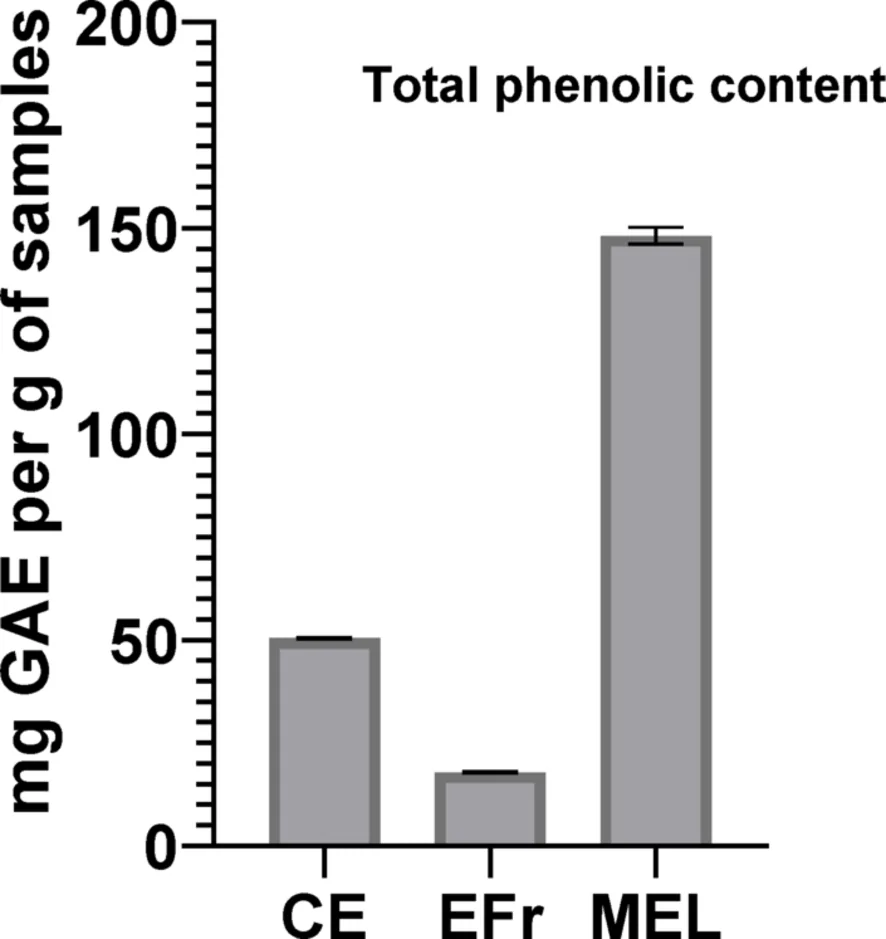
Total phenolic content of crude extract (CE), ethyl acetate fraction (EFr) and methanol fraction (MEL) in mg GAE/g of dry sample

The TPC of the crude extract, and fraction were calculated using the calibration curve equation and reported as mg GAE/g dry mass of the extract. The results are shown in Fig. 6. The TPC were 50.38 ± 0.17; 17.83 ± 0.15; and 148.2 ± 2.02 mg GAE/g for crude extract, EtOAc fraction, and MeOH fraction, respectively. These TPC values were respectively moderate, low and higher [47, 48]. Although numerous phenolic compounds were detected in the ethyl acetate fraction by LC-MS, their overall concentration appears to be relatively low as revealed by the TPC. Conversely, the methanol extract contained a substantially higher level of phenolic constituents of phenolic compounds.

### Antioxidant capacity

**Fig. 7:**
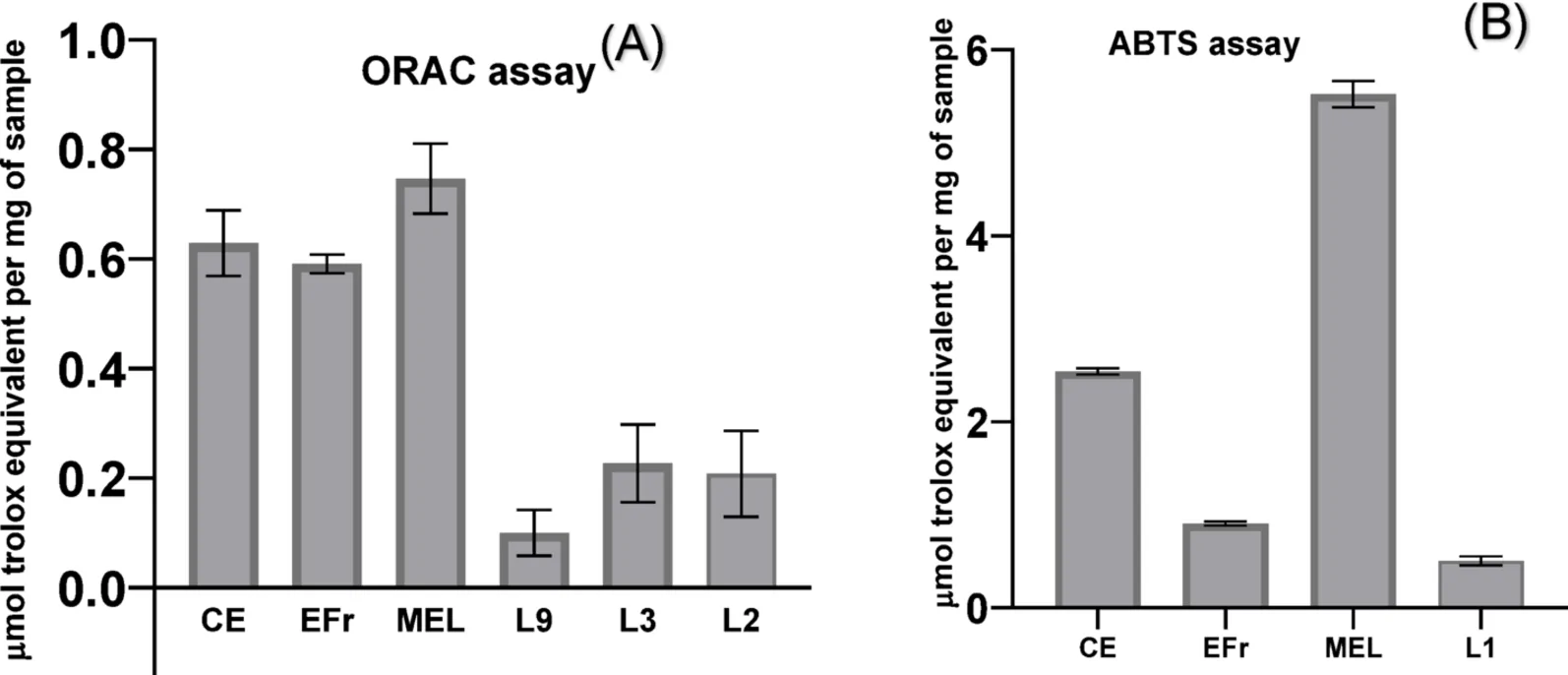
Antioxidant capacity of the crude extract, EtOAc fraction, and isolated compounds assessed both in ORAC (**A**) and ABTS (**B**) assays (initial concentrations of samples 1 mg/mL). CE = crude extract, EFr = ethyl acetate fraction, L9 = compound **1**, L3 = compound **2**, L1 = compound **3**, L2 = compound **4**,

The antioxidant activity of the samples was evaluated using two complementary assays: Oxygen Radical Absorbance Capacity assay (ORAC) and 2,2′-Azino-bis(3-ethylbenzothiazoline-6-sulfonic acid) (ABTS). The crude extract demonstrated strong antioxidant activity in the ABTS assay, with a value of 3.831 ± 0.29 µmol TE/mg (Fig. 7), indicating that 1 mg of extract possesses radical-scavenging capacity equivalent to approximately 3.8 µmol of Trolox (0.9575 mg). However, this activity strongly correlated with the Total Phenolic Content (TPC) results. Thus, correlating the phenolic content to be proportional to the antioxidant capacity of the crude extract and the two fractions.

Among the isolated compounds, only L1 exhibited measurable activity, with a value of 0.5086 ± 0.05 µmol TE/mg (1 mg of L1 has the same activity as 0.125 mg of trolox). This compound had no effect in ORAC assay. The samples exhibited limited proton transfer capacity compared to their electron transfer activity, as only the crude extract and the ethyl acetate fraction displayed activity of approximately 0.6 µmol TE/mg. The superior antioxidant capacity of the methanol fraction can be attributed to its high polyphenol concentration (148.2 ± 2.02 mg GAE/g), which aligns with the predominance of flavonoid compounds identified by LC-MS analysis. Although several phenolic compounds were identified in the ethyl acetate fraction, their concentrations are likely not enough to significantly contribute to the observed antioxidant potential of the extract. This has also been supported by the TPC results.

### Cytotoxicity assay

The cytotoxicity of the crude extract, ethyl acetate fraction, and compounds **1–4** was assessed against human keratinocytes (HaCaT), human melanocytes (NHEM-Ad) Human Embryonic Kidney 293 (HEK 293). The resulting assay data were evaluated for normality using the Shapiro–Wilk test, and, as shown in the graphs (Figure S24), the results satisfied the normality assumption.

**Fig. 8:**
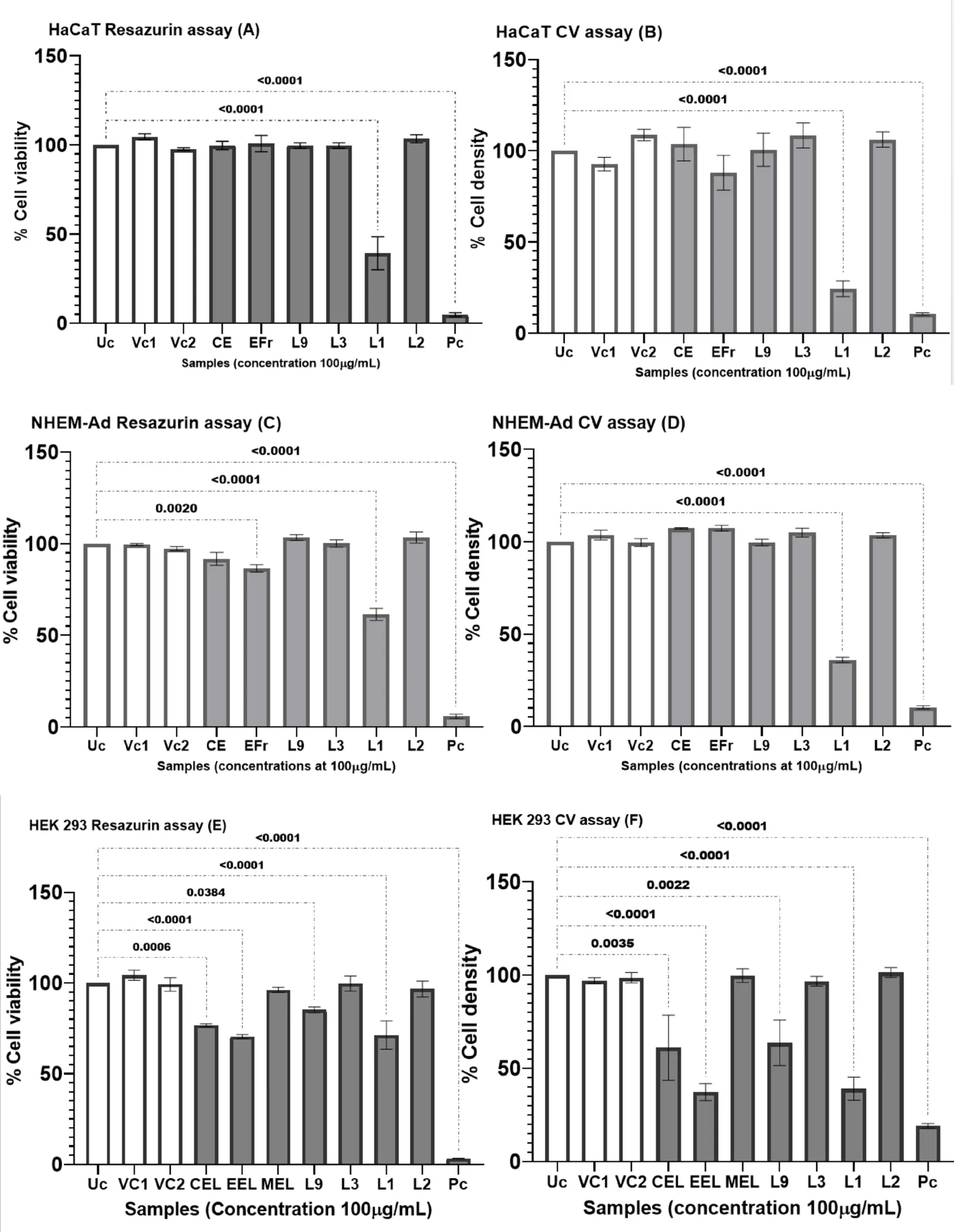
The effect of crude extract, ethyl acetate fraction and isolated compounds **1–4** on the cell number of HaCaT (**A** and **B**), NHEM-Ad (**C** and **D**), and HEK 293 (**E** and **F**) displayed as percentage cell viability and percentage cell density for resazurin and Cristal violet (CV) assays, respectively. Cell number values are expressed as the percentage relative to the complete culture medium. The standard error of the mean is indicated by the error bars. (Uc = Untreated cells, VC = vehicle control, CE = crude extract, EFr = ethyl acetate fraction, L9 = compound **1**, L3 = compound **2**, L1 = compound **3**, L2 = compound **4**, and PC = positive control which is 1% triton X 100)

Ordinary one-way ANOVA analysis indicated that lutein (**3**) significantly reduced the viability of HaCaT, NHEM-Ad, and HEK 293, respectively, to 39.19 ± 9.27, 61.39 ± 3.26, and 71.29 ± 7.82% (*p* < 0.0001) (Fig. 8) compared to the untreated control. The crystal violet indicated subsequent cell densities of 24.35 ± 4.29, 35.96 ± 1.44, and 39.16 ± 6.17% (*p* < 0.0001), relative to the untreated cells. In contrast, the crude extract, the two fractions, and compounds **1**, **2**, and **4** did not affect the viability of HaCaT and NHEM-Ad (Fig. 8). However, these results contrasted with the observations made in the HEK 293 cell line, where the crude extract, ethyl acetate fraction, and compound **1** demonstrated a measurable reduction in cell viability. Specifically, the crude extract, ethyl acetate fraction, and compound 1 decreased cell viability to 76.7 ± 0.83% (*p* = 0.0006), 70.5 ± 1.07% (*p* < 0.0001), and 85.21 ± 1.67% (*p* = 0.0384), respectively. This observation well correlated with the cell density assay. The results are summarized in Fig. 8, which displays the graphs generated using GraphPad Prism, illustrating the effect of each sample on both cell lines.

To the best of our knowledge, no previous studies have reported the toxicity of *Erythrina caffra* leaves. Though Lutein (**3**) affected the cell viabilities, previous reports showed it protect HepG2 cells against tert-butylhydroperoxide mediated suppression of ^14^C-glucose oxidation at 10 µM [49]. Lutein is a widely distributed dietary carotenoid known primarily for its antioxidant and cytoprotective properties. Previous studies have shown that, at concentrations approximately 17-fold lower [49] than the cytotoxic concentration screened in the present study (176 µM), lutein exerts a protective effect against cisplatin-induced oxidative stress in HepG2 cells [50], preserving cellular redox balance and viability [50]. This observation aligns with the general notion that lutein displays low toxicity in hepatic models while acting as an antioxidant. However, to date, no studies have documented its direct cytotoxicity toward human skin-derived cells (keratinocytes or melanocytes), and toward HEK 293 cells. Previous reports have shown that stigmasterol (**2**) exhibits a CC_50_/IC_50_ of 123.0 µg/mL against Vero (E6) cells (a normal mammalian line) [51].

The crude extract, ethyl acetate fraction, and compounds **1**,** 2**, and **4** showed no significant cytotoxicity toward HaCaT and NHEM-Ad cells, indicating a favourable safety profile for skin-derived cells. However, HEK 293 cells exhibited a certain sensitivity to the crude extract, ethyl acetate fraction, and compound **1**, suggesting cell-type–specific effects. This heightened sensitivity may stem from renal cells’ high mitochondrial activity and detoxification role, making them more prone to xenobiotic-induced stress [52]. The consistent results from resazurin and crystal violet assays confirm that the reduced viability reflects genuine cytotoxicity.

### Anti-SARS-CoV-2 activity

The anti-SARS-CoV-2 activity of the extracts, fractions, and the isolated compound is presented in Table 3. Due to limited resources, compound **3** was prioritized for this assay due to its reported multifunctional bioactivity, including antiviral effects against influenza A virus and significant anti-inflammatory potential [53, 54]. In this assay, the reference inhibitor GRL0617 demonstrated the strongest antiviral effect, with an IC₅₀ value of 0.487 µg/mL, thereby validating the reliability and sensitivity of the experimental system. In comparison, all plant-derived samples exhibited markedly lower activity, as indicated by their significantly higher IC₅₀ values. The isolated compound **3** displayed low antiviral potency (IC₅₀ = 389.0 ± 0.35 µg/mL or 683.8 ± 0.6 µmol/L), suggesting that it is unlikely to be the major contributor to the antiviral activity of the extract. These findings indicate that, despite some biological activity observed in other assays, the constituents of these samples do not significantly contribute to anti-SARS-CoV-2 effects.

Overall, none of the tested extracts or isolated compound demonstrated strong antiviral activity when compared to the reference inhibitor GRL0617. The consistently high IC₅₀ values observed across all samples reflect weak inhibition of viral activity, even at relatively high concentrations. The limited activity observed for the ethyl acetate fraction and compound L1 (**3**) may be attributed to the absence or low abundance of compounds capable of effectively targeting the SARS-CoV-2 papain-like protease (PLpro). Although flavonoids and related phenolic compounds have been reported to exhibit antiviral properties, their efficacy is highly dependent on specific structural features, including hydroxylation patterns, prenylation sites, and overall molecular conformation [55, 56].

**Table 3:** Anti-SARS-CoV-2 activity of the plant extracts, fractions and compound EL1

CEL (in µg/mL)	EEL (in µg/mL)	MEL (in µg/mL)	L1 (in µg/mL)	GRL0617
323.6 ± 16.93	258.0 ± 2.48	< 100	389.0 ± 0.35 or 683.8 ± 0.6 µmol/L	0.487 µg/mL or 1.59 µmol/L

CEL-Crude MeOH leave extract of *E. caffra*; EEL- EtOAc fraction of of *E. caffra*; MEL-methanol fraction of *E. caffra*; L1 – compound 3

In a computational investigation against two key SARS-CoV-2 enzymes, namely the main protease (3CLpro) and endoribonuclease (Nsp15), stigmasterol exhibited binding energies of − 6.30 and − 8.22 kcal/mol, respectively, with a particularly low inhibition constant (Ki = 0.942 µM) against Nsp15 [57]. It was identified as a probable inhibitor of SARS-CoV-2 proteins and showed strong binding affinity toward COX-2, a key enzyme involved in post-COVID inflammation, with a docking score of 7.9 kcal/mol [58]. Previous studies have equally shown that Lutein can modulate inflammatory signaling pathways by suppressing pro-inflammatory cytokines, including TNF-α, IL-1β, and IL-6, while reducing oxidative stress. These effects are particularly relevant in the context of SARS-CoV-2 infection, where excessive inflammation and cytokine release contribute significantly to disease severity [59].

The phytochemical and biological investigation of the leaves of *Erythrina caffra* led to the isolation of four compounds, including a novel stigmast-5,22-diene 3-O-(6′-tridecanoate)-β-D-glucopyranoside (erythrinoside), alongside stigmasterol, lutein, and triacontanol. The structures of these compounds were elucidated through comprehensive spectroscopic analyses (¹H, ¹³C, DEPT-135, and 2D NMR) and high-resolution mass spectrometry (HRESIMS). Complementary LC–MS/MS profiling revealed twenty-two additional metabolites, predominantly flavonoids and phenolic derivatives, several of which have previously been reported in the genus *Erythrina*.

The methanol fraction exhibited the highest total phenolic content (148.2 ± 2.02 mg GAE/g) and the strongest antioxidant capacity in both ORAC and ABTS assays, confirming a correlation between phenolic content and radical-scavenging potential. Cytotoxicity assays demonstrated that the crude extract, fractions, and most isolated compounds were non-toxic to human keratinocytes and melanocytes. Only lutein exhibited moderate cytotoxicity at high concentrations, consistent with its known pro-oxidant effects under specific conditions. The anti-SARS-CoV-2 evaluation revealed that *Erythrina caffra* leaf extracts, fractions, and isolated compounds exhibit only weak inhibition of PLpro activity, with no sample approaching the potency of the reference inhibitor. However, the modest activity observed, particularly in the methanol fraction, does not preclude antiviral potential. Given the richness in flavonoids and phenolic compounds, these constituents may exert anti-SARS-CoV-2 effects through alternative mechanisms, such as inhibition of viral entry, modulation of host inflammatory responses, or interference with other viral targets. In silico ADMET analysis suggested that the isolated compounds possess acceptable pharmacokinetic characteristics, including good intestinal absorption and limited toxicity risks, though compound-specific variations were noted.

Overall, these findings highlight *E. caffra* leaves as a promising source of bioactive sterol glycosides, flavonoids, and phenolic antioxidants. The discovery of erythrinoside expands the chemical diversity known within the *Erythrina* genus. Future studies should focus on mechanistic evaluations of antioxidant and cytoprotective pathways, as well as in vivo validation of the pharmacological potential of the polar fractions and the newly identified compound.

## Supplementary Information

Below is the link to the electronic supplementary material.

Supplementary Material 1

## Data Availability

All data generated or analysed during this study are included in this published article and its supplementary information file.
