# Supplementary material for: LC-MS/MS-based phytochemical profiling of Erythrina caffra leaves, isolation of a novel sterol glycoside, and evaluation of antioxidant, cytotoxic, and anti-sars-cov-2 activities
Source: Appl Biol Chem. 2026 Aug 25;69(1):49. doi: 10.1186/s13765-026-01126-w (PMC13506556; doi:10.1186/s13765-026-01126-w)
Supplement: Supplementary file 1 [file 13765_2026_1126_MOESM1_ESM.docx]

**LC-MS/MS-Based Phytochemical Profiling of Erythrina caffra Leaves and Isolation of a Novel Sterol Glycoside with Antioxidant, Cytotoxic, and Anti-SARS-CoV-2 Activities**

Bienvenu Tsakem^a,b,c^, June Cheptoo Serem^b^, Yvette Nkondo Hlophe^c^, Nomusa Sikhakhane^a^, Rémy Bertrand Teponno^d^, Louis Pergaud Sandjo^e^, Xavier Siwe Noundou^a,^*

^a^ Department of Pharmaceutical Sciences, School of Pharmacy, Sefako Makgatho Health Sciences University, PO Box 218 MEDUNSA, Pretoria, 0204, South Africa

^b^ Department of Anatomy, School of Medicine, University of Pretoria, Pretoria, South Africa

^c^ Department of Physiology, School of Medicine, University of Pretoria, Pretoria, South Africa

^d^ Department of Chemistry, Faculty of Science, University of Dschang, Dschang, Cameroon

^e^ Department of Chemistry, Federal University of Santa Catarina, Campus Universitário-Trindade, 88040-900 Florianópolis, SC, Brazil

* Correspondence: Email: xavier.siwenoundou@smu.ac.za, Tel.: +27 12 521 5647 (XSN)

# Abstract

From the crude ethanol extract of Erythrina caffra leaves, a new compound, **stigmasta-5,22-diene 3-O-(6'-tridecanoate)-β-D-glucopyranoside (1)**, along with **stigmasta-5,22-diene (2), lutein (3)**, and **triacontanol (4)**, was isolated. Their structures were elucidated using spectroscopic (1D and 2D NMR) and spectrometric (HRESIMS) analyses. RP-LC-MS/MS profiling of the ethyl acetate and methanol fractions further identified **22 secondary metabolites**, whose proposed structures were consistent with reported fragmentation patterns and literature data. The cytotoxic effects of the crude extract, fractions, and isolated compounds were evaluated on human keratinocytes (HaCaT), melanocytes (NHEM-Ad), and embryonic kidney cells (HEK293) via resazurin and crystal violet assays. **Lutein (3)** at 100 μg/mL significantly reduced cell viability to **39.19 ± 9.27%, 61.39 ± 3.26%, and 71.29 ± 7.82%,** and cell density to **24.35 ± 4.29%, 35.96 ± 1.44%, and 39.16 ± 6.17%,** respectively, for HaCaT, NHEM-Ad, and HEK293. In contrast, the crude extract and ethyl acetate fraction did not notably affect cell viability. Antioxidant evaluation revealed that the **methanol fraction** possessed the strongest activity (**5.52±0.14 μmol TE/mg**) in the ORAC assay, while all isolated compounds and the crude extract exhibited significant peroxyl-radical scavenging activity. Plant-derived extracts and compounds showed weak to moderate inhibition of SARS-CoV-2 PLpro, with only the methanolic fraction exhibiting limited activity (IC₅₀<100 µg/mL), significantly lower than the reference inhibitor. Overall, these findings highlight the **phytochemical diversity and biological potential of** E. caffra **leaves**, emphasizing their antioxidant capacity and selective cytotoxicity. Future work should isolate and characterize the bioactive constituents responsible for the observed antioxidant effects.

**Keywords:** *Erythrina caffra*, cytotoxicity, antioxidant activity, anti-SARS-CoV-2 activity, stigmasta-5,22-diene 3-*O*-(6'-tridecanoate)-β-D-glucopyranoside, LC-MS/MS analysis

**Figure S1. Extraction and purification of *E. caffra* leaves**

| Gallic acid concentration in mg/mL | Absorbance of sample solutions |
| --- | --- |
| 0 | 0 |
| 0.00363636 | 0.069 |
| 0.00727273 | 0.148 |
| 0.0109091 | 0.218167 |
| 0.0145455 | 0.3105 |
| 0.0181818 | 0.374833 |

**Figure S2**. Calibration curve of Gallic acid for TPC

3100-3600 Cm^-1^

2929 Cm^-1^

1740 Cm^-1^

| Wavenumber | PercentTransmittance |
| --- | --- |
| 648.557 | 68.5187 |
| 652.284 | 69.4751 |
| 656.011 | 70.489 |
| 659.739 | 71.4593 |
| 663.466 | 72.3426 |
| 667.193 | 73.3138 |
| 670.921 | 73.7188 |
| 674.648 | 73.2966 |
| 678.375 | 73.4462 |
| 682.103 | 74.9137 |
| 685.83 | 75.7709 |
| 689.558 | 75.8375 |
| 693.285 | 75.7485 |
| 697.012 | 75.5518 |
| 700.74 | 75.404 |
| 704.467 | 75.8916 |
| 708.194 | 75.7533 |
| 711.922 | 75.355 |
| 715.649 | 75.1037 |
| 719.376 | 74.1565 |
| 723.104 | 73.5447 |
| 726.831 | 74.9241 |
| 730.558 | 76.9346 |
| 734.286 | 78.0449 |
| 738.013 | 78.2516 |
| 741.74 | 78.7344 |
| 745.468 | 79.8706 |
| 749.195 | 80.7881 |
| 752.922 | 80.6841 |
| 756.65 | 80.4834 |
| 760.377 | 81.0864 |
| 764.104 | 81.5207 |
| 767.832 | 81.5219 |
| 771.559 | 82.0333 |
| 775.286 | 82.6508 |
| 779.014 | 82.5246 |
| 782.741 | 82.831 |
| 786.468 | 83.855 |
| 790.196 | 84.4259 |
| 793.923 | 84.1459 |
| 797.65 | 84.2638 |
| 801.378 | 84.9778 |
| 805.105 | 85.1653 |
| 808.832 | 84.7676 |
| 812.56 | 84.3023 |
| 816.287 | 84.1452 |
| 820.014 | 84.1236 |
| 823.742 | 84.1935 |
| 827.469 | 84.8494 |
| 831.196 | 86.02 |
| 834.924 | 86.1649 |
| 838.651 | 84.7801 |
| 842.378 | 84.435 |
| 846.106 | 85.4227 |
| 849.833 | 85.0864 |
| 853.56 | 84.8994 |
| 857.288 | 85.2097 |
| 861.015 | 84.8252 |
| 864.742 | 83.5599 |
| 868.47 | 83.0837 |
| 872.197 | 83.5458 |
| 875.924 | 83.842 |
| 879.652 | 84.185 |
| 883.379 | 84.5848 |
| 887.106 | 83.4095 |
| 890.834 | 82.2665 |
| 894.561 | 82.1612 |
| 898.288 | 81.851 |
| 902.016 | 81.3703 |
| 905.743 | 81.5658 |
| 909.47 | 81.3767 |
| 913.198 | 80.1101 |
| 916.925 | 79.6814 |
| 920.652 | 80.5806 |
| 924.38 | 81.1145 |
| 928.107 | 80.7212 |
| 931.835 | 80.2287 |
| 935.562 | 79.7581 |
| 939.289 | 79.4441 |
| 943.016 | 78.8646 |
| 946.744 | 78.5065 |
| 950.471 | 78.303 |
| 954.198 | 77.5353 |
| 957.926 | 76.75 |
| 961.653 | 75.5348 |
| 965.38 | 73.3561 |
| 969.108 | 70.0834 |
| 972.835 | 68.7181 |
| 976.562 | 70.3056 |
| 980.29 | 70.9465 |
| 984.017 | 69.8343 |
| 987.745 | 68.2348 |
| 991.472 | 66.5974 |
| 995.199 | 64.6058 |
| 998.927 | 62.0892 |
| 1002.65 | 59.8574 |
| 1006.38 | 57.1235 |
| 1010.11 | 54.1105 |
| 1013.84 | 52.5771 |
| 1017.56 | 52.6727 |
| 1021.29 | 53.4718 |
| 1025.02 | 54.3318 |
| 1028.75 | 55.3588 |
| 1032.47 | 56.5609 |
| 1036.2 | 56.9783 |
| 1039.93 | 56.3891 |
| 1043.65 | 55.0851 |
| 1047.38 | 53.8145 |
| 1051.11 | 53.4154 |
| 1054.84 | 53.8673 |
| 1058.56 | 54.6253 |
| 1062.29 | 55.1625 |
| 1066.02 | 55.0975 |
| 1069.75 | 54.4898 |
| 1073.47 | 53.8989 |
| 1077.2 | 53.6353 |
| 1080.93 | 54.5507 |
| 1084.66 | 56.3064 |
| 1088.38 | 58.3106 |
| 1092.11 | 60.9592 |
| 1095.84 | 63.6763 |
| 1099.56 | 65.0018 |
| 1103.29 | 64.6424 |
| 1107.02 | 64.4458 |
| 1110.75 | 65.0444 |
| 1114.47 | 66.5348 |
| 1118.2 | 68.6022 |
| 1121.93 | 70.4305 |
| 1125.66 | 71.8013 |
| 1129.38 | 73.2277 |
| 1133.11 | 74.2186 |
| 1136.84 | 74.7808 |
| 1140.57 | 75.8478 |
| 1144.29 | 76.858 |
| 1148.02 | 76.8898 |
| 1151.75 | 75.9089 |
| 1155.47 | 73.5833 |
| 1159.2 | 70.5917 |
| 1162.93 | 68.2247 |
| 1166.66 | 66.5204 |
| 1170.38 | 65.9063 |
| 1174.11 | 66.5216 |
| 1177.84 | 67.6774 |
| 1181.57 | 69.2207 |
| 1185.29 | 71.1359 |
| 1189.02 | 72.828 |
| 1192.75 | 74.1223 |
| 1196.48 | 75.038 |
| 1200.2 | 75.6607 |
| 1203.93 | 76.4401 |
| 1207.66 | 77.6165 |
| 1211.38 | 78.4137 |
| 1215.11 | 78.6568 |
| 1218.84 | 78.9889 |
| 1222.57 | 79.271 |
| 1226.29 | 79.2749 |
| 1230.02 | 79.1272 |
| 1233.75 | 78.7678 |
| 1237.48 | 78.2363 |
| 1241.2 | 78.1002 |
| 1244.93 | 77.9111 |
| 1248.66 | 77.6284 |
| 1252.39 | 77.8243 |
| 1256.11 | 77.9405 |
| 1259.84 | 77.9623 |
| 1263.57 | 78.3114 |
| 1267.29 | 78.6267 |
| 1271.02 | 78.5672 |
| 1274.75 | 78.5733 |
| 1278.48 | 78.6384 |
| 1282.2 | 78.4784 |
| 1285.93 | 78.2045 |
| 1289.66 | 77.9438 |
| 1293.39 | 77.6938 |
| 1297.11 | 77.8815 |
| 1300.84 | 78.7743 |
| 1304.57 | 79.6737 |
| 1308.3 | 80.0607 |
| 1312.02 | 80.0451 |
| 1315.75 | 79.6822 |
| 1319.48 | 79.2162 |
| 1323.2 | 79.0654 |
| 1326.93 | 79.3904 |
| 1330.66 | 79.9078 |
| 1334.39 | 80.3624 |
| 1338.11 | 80.6947 |
| 1341.84 | 80.8788 |
| 1345.57 | 80.6556 |
| 1349.3 | 79.9748 |
| 1353.02 | 79.0206 |
| 1356.75 | 78.1648 |
| 1360.48 | 77.3932 |
| 1364.21 | 75.9307 |
| 1367.93 | 74.995 |
| 1371.66 | 75.2123 |
| 1375.39 | 75.2631 |
| 1379.12 | 75.7787 |
| 1382.84 | 77.5695 |
| 1386.57 | 80.5999 |
| 1390.3 | 83.9153 |

**Figure S3**. FT-IR spectrum of compound **1**

**Figure S4. LC-MS spectrum of compound 1**

**Figure S5**. HRESIMS(+) spectrum of compound **1**

**Figure S6**. ^1^H NMR (CDCl_3_, 500 MHz) spectrum of compound **1**

**Figure S7**. ^13^C NMR (CDCl_3_, 125 MHz) spectrum of compound **1**

**Figure S8**. DEPT 135 spectrum of compound **1**

**Figure S9**. HSQC spectrum of compound **1**

**Figure S10**. HMBC spectrum of compound 1

**Figure S11.** HRESIMS/MS(+) spectrum of a fragment of Compound **1** at 169.2005 (C_12_H_25_^+^)

**Figure S12**. COSY spectrum of compound 1

**Figure S13.** HRESIMS/MS(+) spectrum of a fragment of Compound **1** at 523.3629 (C_30_H_51_	O_7_^+^)

**Figure S14.** HRESIMS/MS(+) spectrum of a fragment of Compound **1** at 505.3136 (C_27_H_46_	O_7_Na^+^)

**Figure S15.** Fragmentation of compound 1

**Figure S16**. ^1^H NMR (CDCl_3_, 400 MHz) spectrum of compound **2**

**Figure S17**. ^1^H NMR (CDCl_3_, 400 MHz) spectrum of compound **3**

**Figure S18**. ^1^H NMR (CDCl_3_, 400 MHz) spectrum of compound **4**

**Figure S19.** Base Peak Intensity (BPI) of ethyl acetate extract of *E. caffra* leaves HRESIMS(-)

**Figure S20.** Base Peak Intensity (BPI) of ethyl acetate extract of *E. caffra* leaves, HRESIMS(+)

**Figure S21.** Base Peak Intensity (BPI) of MeOH extract of *E. caffra* leaves, HRESIMS(-)

**Figure S22a**. Fragmentation mechanism of Isovitexin (1a) in HRESIMS/MS

**Figure S22b**. Fragmentation mechanism of Chlorogenic acid (2a) in HRESIMS/MS

**Figure S22c**. Fragmentation mechanism of 2,3-Dehydrokievitone (3a)in HRESIMS/MS

**Figure S22d**. Fragmentation mechanism of Naringenin (5a) in HRESIMS/MS

**Figure S22e**. Fragmentation mechanism of 6-prenylapigenin (6a) in HRESIMS/MS

**Figure S22f**. Fragmentation mechanism of Erythrivarone A (7a) in HRESIMS/MS

**Figure S22g**. Fragmentation mechanism of Carpachromene (8a) in HRESIMS/MS

**Figure S22h**. Fragmentation mechanism of gancaonins Q (9a) in HRESIMS/MS

**Figure S22i**. Fragmentation mechanism of Acacetin (10a) in HRESIMS/MS

**Figure S22j**. Fragmentation mechanism of abyssinone V 4’-methyl ether (11a) in HRESIMS/MS

**Figure S19k**. Fragmentation mechanism of Apigenin-7,4'-dimethyl ether (12a) in HRESIMS/MS

**Figure S22l**. Fragmentation mechanism of 2,2'-methylenebis[6-ter-butyl-4-methylphenol] (12a) in HRESIMS/MS

**Figure S22m**. Fragmentation mechanism of Lutein (14a = 3) in HRESIMS/MS

**Figure S22n**. Fragmentation mechanism of β-Carotene 5,6-epoxide (15a) in HRESIMS/MS

**Figure S22o**. Fragmentation mechanism of stigmasterol (16a = 2) in HRESIMS/MS

**Figure S23a**. Fragmentation mechanism of Lucenin III (17a) in HRESIMS/MS

**Figure S23b**. Fragmentation mechanism of Genistein 4,7'-diglucopyranoside (18a) in HRESIMS/MS

**Figure S23c**. Fragmentation mechanism of Isoschaftoside (19a) in HRESIMS/MS

**Figure S23d**. Fragmentation mechanism of Luteolin-6-*C-β-D*-glucopyranoside (20a) in HRESIMS/MS

**Figure S23e**. Fragmentation mechanism of Vitexin (21a) in HRESIMS/MS

**Figure S23f**. Fragmentation mechanism of 1-O-(E)-cinnamoyl-4-galloyl-b-D-glucopyranose (22a) in HRESIMS/MS

**Figure S24**. Normality graphs in Shapiro-Wilk test, A = HaCaT resazurin assay, B = HaCaT CV assay, C = NHEM-Ad Resazurin assay, D = NHEM-Ad CV assay, E = HEK 293 Resazurin assay, and D = HEK 293 CV assay

## Pharmacokinetic parameters of the isolated compounds

The Absorption, Distribution, Metabolism, Excretion, and Toxicity (ADMET) properties of compounds **1-4** were simulated from ADMETlab3.0 (<https://admetlab3.scbdd.com>). Compounds **2-4** are well absorbed by intestines [55] as indicated by HIA (Human intestinal absorption) and MDCK (Madin-Darby canine kidney. As Caco-2 and Madin-Darby Canine Kidney (MDCK) monolayer assays are popularly used to assess the passive permeability of small molecules *in vitro*, compounds **2-4** are predicted to be well absorbed passively. Parallel Artificial Membrane Permeability Assay (PAMPA) ( is also associated to good absorption but at a pH within 5.5 to 7.4 [55]. The volumes of distribution of **1-3** are estimated to be respectively 0.283, 0.805, and 1.131 L/Kg while compound **4** has a poor distribution. Compound **3** has low neurotoxicity and demonstrates good blood-brain barrier (BBB) permeation, medium fraction unbound to plasma (Fu, 7.068), making it a promising candidate for the treatment of brain disorders. Unfortunately, these compounds appeared to cause insignificant damage to the liver with a DILI (Drug Induced Liver Injury) value of 0.296 and many other toxicity parameters are associated. Other compounds expressed numerous toxicities such as nephrotoxicity, neurotoxicity, hematotoxicity, genotoxicity, and immunotoxicity. All these compounds have medium excretion via plasma, excepted compound **4,** which has excellent excretion. Table **4** contains the ADMET properties of compounds **1-4**.

**Table 4**. Absorption, Distribution, Metabolism, Excretion, and Toxicity properties of compounds **1-4**

|  | **1** | **2** | **3** | **4** |
| --- | --- | --- | --- | --- |
| Absorption | MDCK permeability, PAMPA, Pgp inhibitor and substrate, HIA | Caco-2 permeability (-5.075), MDCK permeability, PAMPA (0.006), Pgp substrate, HIA ≥ 30% | Caco-2 (-5.077) and MDCK permeability, HIA ≥ 30% | Caco-2 permeability (-5.099), MDCK permeability, PAMPA, Pgp substrate |
| Distribution | VDss (1.759) and BBB | VDss (0.805) and BBB | VDss (0.053), BBB (0.0), and Fu (7.068) | BBB and OATP1B1 |
| Metabolism | CYP2B6, CYP2C8 and HLM stability | CYP2C8 and HLM stability | CYP2B6, CYP2C8 and HLM stability | CYP2C9, and CYP2D6 substrates, CYP2B6, and CYP2C8 inhibitors |
| Excretion | CL_plasma_ (2.95 mL/min/kg) | CL_plasma_ (12.944 mL/min/kg) | CL_plasma_ (9.574 mL/min/kg) | CL_plasma_ (4.474 mL/min/kg) |
| Toxicity | AMES toxicity (0.03), rat oral acute toxicity (0.035), FDAMDD (0.283), eye corrosion and irritation (0.004), neurotoxicity (0.001), hematotoxicity (0.066), genotoxicity and immunotoxicity (0.134) | hERG blockers (0.261), DILI (0.111), AMES toxicity (0.049), rat oral acute toxicity (0.209), skin sensitization (0.29), eye corrosion (0.004) and irritation (0.147), nephrotoxicity (0.107), neurotoxicity (0.202), hematotoxicity (0.048), genotoxicity (0.004) and immunotoxicity (0.091) | hERG blockers (0.104 µM), DILI (0.296), eye irritation (0.296), immunotoxicity (0.164) | DILI (0.082), AMES (0.009), rat oral acute toxicity (0.071), FDAMDD (0.205), carcinogenicity (0.184), nephrotoxicity (0.09), neurotoxicity (0.002), ototoxicity (0.134), hematotoxicity, (0.091), immunotoxicity (0.031), and Hek293 cytoxicity (0.24) |

HLM :Human Liver Microsomal, CL_plasma_ :plasma clearance, FDA Maximum Recommended Daily Dose (FDAMDD):Food and Drug Administration Maximum Daily Dose, hERG:human ether-a-go-go related gene
